# Gunshot Airborne Surveillance with Rotary Wing UAV-Embedded Microphone Array

**DOI:** 10.3390/s19194271

**Published:** 2019-10-01

**Authors:** Felipe Gonçalves Serrenho, José Antonio Apolinário, António Luiz Lopes Ramos, Rigel Procópio Fernandes

**Affiliations:** 1 Department of Electrical Engineering, Military Institute of Engineering (IME), Rio de Janeiro 22290-270, Brazil; apolin@ime.eb.br; 2 Department of Science and Industry Systems, University of South-Eastern Norway (USN), 3616 Kongsberg, Norway; antonio.ramos@usn.no; 3 Program of Defense Engineering, Military Institute of Engineering (IME), Rio de Janeiro 22290-270, Brazil; rigelfernandes@gmail.com

**Keywords:** unmanned aerial vehicles (UAV), rotary wing drones, direction of arrival (DoA) estimation, microphone array, gunshot audio surveillance, shooter localization

## Abstract

Unmanned aerial vehicles (UAV) are growing in popularity, and recent technological advances are fostering the development of new applications for these devices. This paper discusses the use of aerial drones as a platform for deploying a gunshot surveillance system based on an array of microphones. Notwithstanding the difficulties associated with the inherent additive noise from the rotating propellers, this application brings an important advantage: the possibility of estimating the shooter position solely based on the muzzle blast sound, with the support of a digital map of the terrain. This work focuses on direction-of-arrival (DoA) estimation methods applied to audio signals obtained from a microphone array aboard a flying drone. We investigate preprocessing and different DoA estimation techniques in order to obtain the setup that performs better for the application at hand. We use a combination of simulated and actual gunshot signals recorded using a microphone array mounted on a UAV. One of the key insights resulting from the field recordings is the importance of drone positioning, whereby all gunshots recorded in a region outside a cone open from the gun muzzle presented a hit rate close to 96%. Based on experimental results, we claim that reliable bearing estimates can be achieved using a microphone array mounted on a drone.

## 1. Introduction

The interest in automatic sniper localization systems traces back to the early 1990s, pioneered by countries such as the United States of America, Russia, Canada, France, and more recently, Israel, among others. Such surveillance systems for shooter detection and localization can be useful to the police and military forces [1,2]. The shooter detection and localization problem can be approached in different ways, depending on the kind of signatures from a gunshot event, acoustic or electromagnetic, that one decides to process [3]. For instance, cameras can be used to detect the muzzle flash [4], whereas microphone arrays can be used to detect the muzzle blast and the shockwave acoustic signatures. If these two acoustic signatures are detected in the same gunshot event, one can estimate the location of the shooter using a two-step procedure [3].

The successful use of microphone arrays to tackle the direction-of-arrival (DoA) estimation problem even with low signal-to-noise ratio (SNR) can be seen in Reference [5]. In this paper, median filtering is used to enhance the collected acoustic gunshot signals. In Reference [6], an algorithm that optimizes DoA estimation using exhausive search through consistent fundamental loops is introduced. This method, in an attempt to have the best approach for the level of noise of audio signals, is a combination of standard DoA estimation, Exhaustive Search (ES) [7], and consistent fundamental loop [6].

Microphone arrays can be deployed in different platforms, e.g., stand-alone systems mounted on vehicles [8], on light posts in urban areas or on trees in a forest [9]. All these systems are currently subjects of great interest in academia and are recently associated with the internet of things (IoT) industry [10] as well. However, one such system based on a microphone array mounted on an aerial drone brings additional advantages owing to its flexibility to cover wider areas relatively quicker and at a lower cost. It also opens the opportunity for new important applications, such as search-and-rescue missions [11,12] and environmental monitoring [9]. In Reference [11], a microphone array mounted on a drone is used to detect a narrowband signal generated by a whistle, which can be very effective in search-and-rescue missions in areas of difficult access.

An application example for environmental monitoring is presented in Reference [9], suggesting the use of an open hardware to be deployed in the forest to record audio signals and using a Secure Digital (SD) card to store the data. These signals vary from bat ultrasounds to gunshot signals. For instance, in the case of detecting gunshot events in protected areas, people responsible for monitoring those areas could be triggered to carry out necessary actions against poaching. Hoshiba et al. presented detailed design and implementation of a quadcopter-embedded microphone array system for outdoor environments [12].

In order to enable new drone applications, the scientific community has developed an interest for new techniques capable of tackling the strong ego-noise presented in audio recordings from unmanned aerial vehicle (UAV)-embedded microphone arrays, especially when the target sound is a whistle or human speech. Methods based on Multiple Signal Classification (MUSIC), known to be very robust against noise, are presented in Reference [13]. The Generalized Eigenvalue Decomposition-MUSIC (GEVD-MUSIC) [14,15] is reported to have high performance even for low-SNR signals. The incremental Generalized Eigenvalue Decomposition-MUSIC (iGEVD-MUSIC) introduced in Reference [16] estimates the noise correlation matrix incrementally to cope with the high non-stationarity of the ego-noise. A supervised approach that uses UAV sensors data and motor rotation speeds to estimate the noise correlation matrix was proposed in Reference [17]. Aiming at reducing computational complexity and errors associated with inaccuracies in noise correlation matrix estimation, Reference [18] proposes the Multiple Signal Classification based on incremental Generalized Singular Value Decomposition-MUSIC (iGSVD-MUSIC) with Correlation Matrix Scaling (CMS).

A novel algorithm to sound source location with UAV-embedded microphone arrays based on time-frequency bins was proposed in Reference [19]. This method takes advantage of the fact that ego-noise and target sound (e.g., speech or emergency whistle) mainly consist of harmonic components that usually occupy different time-frequency bins. In Reference [20], the time-frequency technique is associated with a time-frequency spatial filter to enhance the signal of interest. Other interesting researches related to sound processing with drones include the following: a study about the ego-noise of multirotors micro aerial vehicles [21], that also proposes the use of Blind Source Separation (BSS) algorithm to suppress it; the use of ego-noise to measure the relative directions between multirotors in a swarm [22]; and the ability to track moving sound sources [23].

Focusing on gunshot airborne surveillance, the deployment of acoustic sensors in elevated platforms could enable advantages for shooter-position estimation, according to Reference [24]. The use of an aerial drone as a mobile elevated platform was investigated in Reference [25], using only simulations. Different noise levels were synthesized using drone noise recordings in a silent room and real gunshot signals recorded with a high-quality microphone mounted on a tripod in an open field. Also in this work, signal enhancement techniques were employed along with DoA estimation algorithms and target motion analysis was used to estimate the shooter’s position. In Reference [26], the geometry deployment of the microphone array is discussed, taking into account the wind produced by the propellers, the electromagnetic interference, and the scarce space available on the drone.

In this paper, we focus on the details of DoA estimation of gunshot signals obtained from a microphone array aboard a flying drone. We used simulations to investigate the performance of preprocessing and DoA estimation techniques, also tuning theirs parameters. The most appropriated methods were evaluated with actual field recordings, given the position and attitude of the drone obtained from its GPS and inertial unit.

The rest of this paper is organized as follows. Section 2 starts with a brief overview on gunshot acoustics, followed by a discussion on the techniques used in a UAV-based gunshot surveillance system, namely signal preprocessing, gunshot detection, DoA estimation, and shooter localization. Section 3 describes the hardware used and the shooting site as well as how telemetry data is recovered and used, while Section 4 discusses experimental results from simulations and from actual gunshot signals collected using an array of sensors mounted on a flying drone. The discussion and conclusion are addressed in Section 5.

## 2. DoA Estimation and Shooter Localization

The acoustic signatures generated by a gunshot event can be divided into three parts, namely the muzzle blast, the shockwave, and sounds related to mechanical actions, which include the trigger and hammer mechanisms, ejection of spent cartridges, and the loading system. The mechanical action-related sounds can be useful in forensics analysis [27,28]. However, they are of no interest in the design of sniper localization systems, since they can only be recorded using sensors placed close to the gun.

The muzzle blast is generated by the expansion of gases in the gun barrel and is louder in the direction the barrel is pointing toward [29,30]. It propagates at the speed of sound and lasts typically from 3 to 5 ms [31]. The energy of the muzzle blast depends on the firearm used, and it is almost always audible in a given range, provided that silencers or suppressors are not used [31,32].

**Figure 1:**
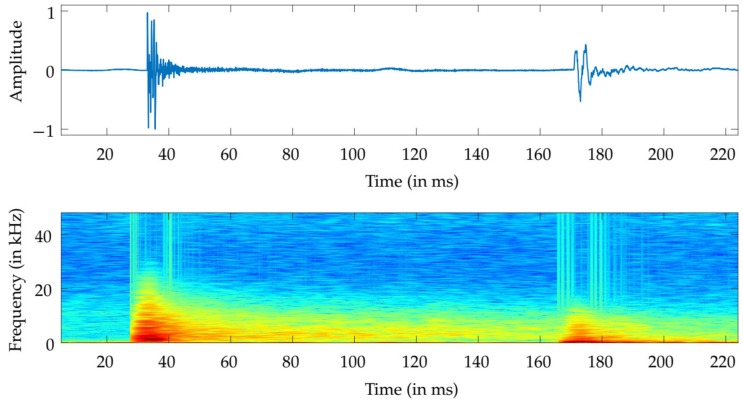
Components of a gunshot signal: shockwave (left) and muzzle blast (right) of a caliber 7.62 mm rifle and the corresponding spectrogram.

A shockwave will be generated for as long as a projectile is travelling faster than the speed of sound and propagates outwards from the bullet trajectory at an angle known as the Mach angle [33]. The shockwave generated by a typical supersonic bullet lasts for approximately 200 µs, and its frequency spectrum has a wider frequency band than that of the muzzle blast, as exemplified in Figure 1. Since the shockwave propagates in a cone shape following the bullet trajectory, it cannot be detected if the bullet is moving away from the position where the sensors are located [34]. This constitutes a problem for shooter localization systems that rely on the detection of both shockwave and muzzle blast signals.

The shooter’s localization problem may be divided in four steps, namely preprocessing, gunshot detection and muzzle blast identification, DoA estimation, and shooter-position estimation.

### 2.1. Preprocessing

Localization of a shooter based on the audio acquired from a drone is especially challenging due to the presence of strong ego-noise, mainly generated by the propellers [35]. This becomes an even greater challenge under long-range shootings, whereby the detection and the direction-of-arrival estimation of the muzzle blast signal is compromised.

When signals of interest are approximately stationary, such as tiny voice snippets, whistles, or white noise, methods based on noise correlation matrix estimation, such as Wiener Filtering [36], are used. For impulsive signals, as in the case of gunshot signals, median filtering is an alternative option [5]. In this work, we evaluate the performance of these methods in the task of DoA estimation.

During steady parts of a flight, where stationarity can be assumed [36], we may use Wiener filtering to attenuate the influence of the ego-noise. We used in this work the implementation developed by Liu Ming and Pascal Scalart [37,38]. This Wiener filter, referred to as Two-Step Noise Reduction (TSNR), uses the decision-directed approach [39] to track a priori SNR and refines the SNR estimation to avoid reverberation effects.

Median filtering was employed in Reference [40] as a technique to separate percussive and harmonic components of a signal. The proposed scheme uses the concept that percussive components can be seen as outliers in the time domain while harmonic sounds can be seen as outliers in the frequency domain. Median filtering, described next, is capable of removing these outliers and of separating these different acoustic signatures to some extent. Given the input x(k), the output y(k) is the median value of the window with length Δ centered in x(k). The parameter should be chosen accordingly, in such a way that the expected duration of the artifacts is removed but without significant impact on the signal of interest. The median filtering can be expressed according to the following:

(1) y(k)=median{x(k−l:k+l)},l=(Δ−1)/2,ifΔis odd;

if Δ is even, the median is defined as the mean of the two central median values.

The use of median filtering to estimate background noise embedded in gunshot signals was introduced in Reference [5], which computes the enhanced signal as s(k)=x(k)−y(k). To preserve the muzzle blast’s shape, Δ should not represent less than half of its duration, which is approximately 3 ms [31].

### 2.2. Gunshot Detection

As previously noted, a gunshot surveillance system must be able to detect an impulsive signal and to identify if it is a muzzle blast component, a shockwave component, or none of them. There is a vast literature available about this matter [41,42,43,44,45,46].

The method in Reference [41] uses a transient detection, introduced in Reference [42], that looks for significant changes in the signal energy. For muzzle blast and shockwave classification, Reference [41] uses two tests: the first one is based on the spectral information of the signal, and the second one uses time difference of arrival between neighboring peaks.

A detection scheme based on correlation against a template is proposed in Reference [43], where the authors claim that the method could be implemented by a low power consuming hardware. Correlation against a template is also addressed in Reference [44], where it is compared against classical algorithms usually used in speech processing; their results conclude that correlation matches the performance of those algorithms, especially in noisy environments. In Reference [45], linear predictive coding (LPC) coefficients are combined with template matching to increase the performance of gunshot detection systems, especially regarding false-positive errors.

A wavelet-based approach [46] can be used to distinguish three acoustic events: muzzle blast, shockwave, and reflections. Furthermore, according to the authors, this method can classify the caliber based on the muzzle blast or on the shockwave signals.

The strong ego-noise of a drone is not white and is highly nonstationary [36]. Furthermore, it is strongly dependent on the drone used and on the positioning of the sensors. These are additional challenges to detection and muzzle blast–shockwave classification. These tasks were carried out manually in this work.

### 2.3. DoA Estimation Methods

In this section, we first define DoA angles and then present two DoA estimation methods: a data selection least squares method [7] and an angular spectrum-based method named the Multi-channel Blind Source Separation (MBSS) Locate [47].

**Figure 2:**
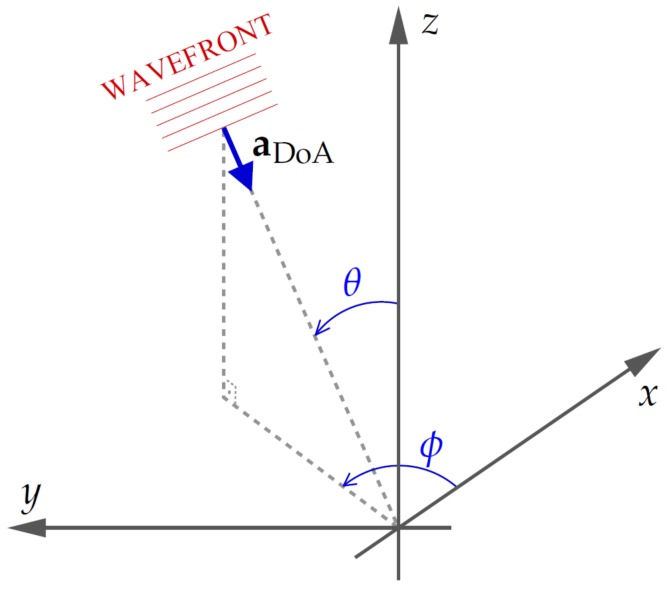
Azimuth (ϕ) and zenith (θ) relative to the center of the array: The *x* axis is oriented to the front of the drone, and the *z* axis points upwards.

Figure 2 shows the angles (azimuth ϕ and zenith θ) that define the DoA. It is noteworthy that the azimuth herein is taken counterclockwise, as in Reference [48]. Thus, the unit vector in the direction of sound wave propagation is given as follows:

(2) $$a_{\mathrm{DoA}}={\left[-\cos(\phi )\sin(\theta )-\sin(\phi )\sin(\theta )-\cos(\theta )\right]}^{T}.$$

#### 2.3.1. The Data Selection Least Squares DoA Estimation Algorithm

The first step of the least squares (LS) method is the time-delay estimation (TDE) between the sensor pairs in the array. Next, we use an LS cost function associated with a data-selective algorithm. The TDEs are obtained from the peak of the cross-correlation $r_{x_{i}x_{j}}\left(\tau \right)$ defined as follows [49]:

(3) $$r_{x_{i}x_{j}}\left(\tau \right)=E\left[x_{i}\left(k\right)x_{j}\left(k-\tau \right)\right],$$

where E denotes the expectation operator and τ is the lag between two given sensors, $x_{i}$ and $x_{j}$. In practice, we do not have statistical knowledge of the signals, and Equation (3) is usually approximated by its time average given by the following:

(4) $${\hat{r}}_{x_{i}x_{j}}\left(\tau \right)=\sum_{k=-\infty }^{\infty }x_{i}\left(k\right)x_{j}\left(k-\tau \right)=x_{i}\left(\tau \right)*x_{j}\left(-\tau \right),$$

where * is the convolution operator.

Taking the discrete Fourier transform of ${\hat{r}}_{x_{i}x_{j}}\left(\tau \right)$ and assuming real-valued signals, we can write the cross power spectrum density between $x_{i}\left(k\right)$ and $x_{j}\left(k\right)$ as follows:

(5) $${\hat{R}}_{x_{i}x_{j}}\left(e^{jw}\right)=F\left\{{\hat{r}}_{x_{i}x_{j}}\left(\tau \right)\right\}=F\left\{x_{i}\left(\tau \right)*x_{j}\left(-\tau \right)\right\}=X_{i}\left(e^{jw}\right)X_{j}\left(e^{-jw}\right)=X_{i}\left(e^{jw}\right)X_{j}^{*}\left(e^{jw}\right).$$

The cross-correlation can then be computed using the following:

(6) $${\hat{r}}_{x_{i}x_{j}}\left(\tau \right)=F^{-1}\left\{{\hat{R}}_{x_{i}x_{j}}\left(e^{jw}\right)\right\}.$$

Adding a frequency weighting function in Equation (6), we have the generalized cross-correlation (GCC) as follows:

(7) $${\hat{r}}_{x_{i}x_{j}}\left(\tau \right)=F^{-1}\left\{\psi \left(w\right){\hat{R}}_{x_{i}x_{j}}\left(e^{jw}\right)\right\},$$

where classical cross-correlation corresponds to ψ(w)=1,∀w. A popular weighting scheme employed by the GCC is the phase transform (PHAT) [7,36,49,50,51,52,53], known to have good performance in reverberating scenarios [49]. PHAT also tends to have a sharper peak than classical GCC, increasing the performance of the TDE [50]. The PHAT weighting function is given by the following [53]:

(8) $$\psi^{\mathrm{PHAT}}\left(w\right)=\frac{1}{|X_{i}\left(e^{jw}\right)X_{j}\left(e^{-jw}\right)|}.$$

**Figure 3:**
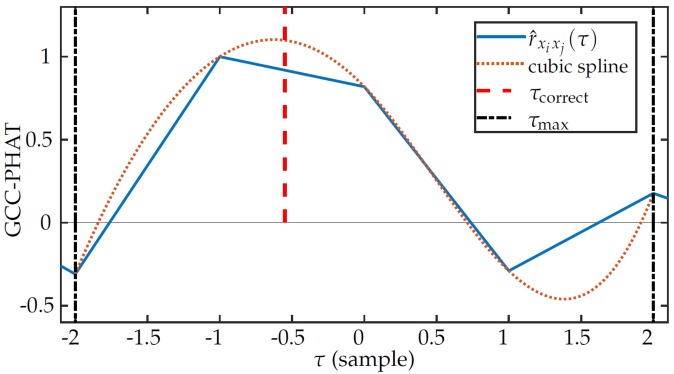
Effect of interpolation in generalized cross-correlation (GCC)-phase transform (PHAT): Note that ${\hat{r}}_{x_{i}x_{j}}\left(\tau \right)$ is the GCC-PHAT without interpolation.

Finally, the TDE is obtained as follows:

(9) $${\hat{\tau }}_{ij}=\underset{\left|\tau \right|\leq \tau_{\max}}{arg max}\left|{\hat{r}}_{x_{i}x_{j}}^{\mathrm{PHAT}}\left(\tau \right)\right|,$$

where $\tau_{\max}$ is the maximum delay possible (in number of samples) between microphone *i* and *j*, which occurs when the DoA has the same direction of the vector that connects sensors *i* and *j*:

(10) $$\tau_{\max}=\frac{|p_{i}-p_{j}|f_{s}}{v_{s}},$$

where $p_{i}$ and $p_{j}$ are the position vectors of sensors *i* and *j*, $v_{s}$ is the speed of sound, and $f_{s}$ is the sampling frequency. The TDEs using inverse Fourier transform (iFFT) provide delays as integer multiples of the sampling period; this leads to errors that are particularly relevant in small arrays (small time delays between sensors) and with low sampling frequency. To mitigate this source of errors, we can interpolate the GCC, allowing more accurate estimations of the time difference of arrival (TDoA). In this work, we used cubic interpolation [54], calculated at every point in between $-\tau_{\max}$ and $\tau_{\max}$, ensuring that all possible values of delay are covered. Figure 3 shows the effect of cubic interpolation over GCC-PHAT in a small array for a signal with $f_{s}=8$ kHz.

**Figure 4:**
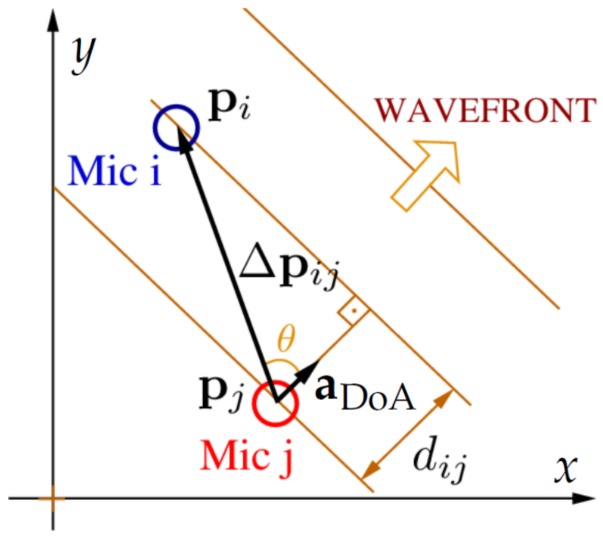
Direction of arrival (DoA) calculation in a 2-D scenario.

Figure 4 illustrates in 2-D the delay between microphones *i* and *j*. In a 3-D scenario, we write $d_{ij}=\Delta p_{i,j}^{T}a_{\mathrm{DoA}}$ such that the TDE (in samples) is given by the following:

(11) $$\tau_{i,j}=\frac{f_{s}{\left(p_{i}-p_{j}\right)}^{T}a_{\mathrm{DoA}}}{v_{s}}=\frac{f_{s}\Delta p_{i,j}^{T}a_{\mathrm{DoA}}}{v_{s}}=\Delta {\bar{p}}_{i,j}^{T}a_{\mathrm{DoA}},$$

where $\Delta {\bar{p}}_{i,j}=f_{s}\Delta p_{i,j}/v_{s}$.

Based on the estimated delay, as given in Equation (9), and the delay based on the unknown vector $a_{\mathrm{DoA}}$, Equation (11), we define the least squares cost function:

(12) $$\xi =\sum_{i,j}{\left(\tau_{i,j}-\Delta {\bar{p}}_{i,j}^{T}a_{\mathrm{DoA}}\right)}^{2},$$

for all possible pairs, N=M(M−1)/2 for the case of *M* microphones.

Minimizing the cost function with respect to $a_{\mathrm{DoA}}$, we find the following:

(13) $$a_{\mathrm{DoA}}=R^{-1}d,$$

where $d=\Delta {\bar{p}}^{T}\tau$, $\tau ={\left[\tau_{1,2}\tau_{1,3}\ldots \tau_{1,N}\tau_{2,3}\ldots \tau_{M-1,M}\right]}^{T}$ and $R=\Delta {\bar{p}}^{T}\Delta \bar{p}$, $\Delta \bar{p}$ are assembled as follows:

(14) $$\Delta \bar{p}={\left[\begin{array}{c} \Delta {\bar{p}}_{1,2}\;\Delta {\bar{p}}_{1,3}\;\ldots \;\Delta {\bar{p}}_{1,M}\;\Delta {\bar{p}}_{2,3}\;\ldots \;\Delta {\bar{p}}_{M-1,M} \end{array}\right]}^{T}.$$

The solution provided by Equation (13) may not have unit norm, which must be ensured through normalization. Only then could azimuth and zenith be calculated using trigonometric operations, according to Equation (2).

Equation (13) provides all three coordinates only when using a spatial array. If a planar array is used, ambiguity occurs and matrix R is singular. When all sensors are in a plane (xy-plane for instance), we must adapt the sensor positions ($p_{i}$) to suppress the coordinate associated with the perpendicular axis, in our case *z*. This way R is non-singular and Equation (13) provides ${\hat{a}}_{\mathrm{DoA}}^{\mathrm{incomplete}}={\left[a_{x}a_{y}\right]}^{T}$. As the $a_{\mathrm{DoA}}$ must be unitary and assuming that the source is located above or below the array, it is possible to estimate the DoA.

The strong ego-noise could compromise the TDEs, generating outliers that would adversely affect the DoA estimation. As for the cost function defined in Equation (12), the solution can be obtained without using all available pairs of microphones; it is possible to use a data-selective algorithm to remove outliers. Using the Exhaustive Search algorithm ES(*n*) [7], we choose the *n* combination from the set of *N* pairs of microphones that minimizes the cost function ξ in Equation (12). We need to be cautious when choosing the number of pairs of microphones to be used, parameter “*n*” in ES(*n*), once it can generate ill-conditioned matrices [55]. The appropriate choice for “*n*” can be obtained according to a decision tree as done in Reference [6].

#### 2.3.2. The MBSS Locate

The Multi-channel Blind Source Separation (MBSS) Locate [56] is available as a MATLAB${}^{®}$ toolbox. It estimates the direction of arrival of multiple sources from audio signals collected by an acoustic sensor array. This software implements multichannel versions of four state-of-the-art and three proposed SNR-based local angular spectra methods for audio signals [47].

The state-of-the-art local angular spectra methods are GCC-PHAT [49] and its version with a nonlinear function GCC-NONLIN [57], Multiple Signal Classification (MUSIC) [13], and Cumulative State Coherence Transform (cSCT) [58]. These techniques, except the cSCT method, rely on the assumption that one source is predominant in each time-frequency bin. The cSCT method assumes that there are at most two predominant sources.

The SNR-based local angular spectra tackles the multisource TDoA estimation problem. The main idea is to use the SNR as an unbounded measure to estimate if the information of a time-frequency bin results from a single source. Blandin et al. [47] proposed three methods to estimate the SNR using two microphones and the following techniques: Minimum Variance Distortionless Response (MVDR) [59], Diffuse Noise Model (DNM) [60], and Minimum Variance Distortionless Response Weighted (MVDRW).

The MBSS full version enables the user to simulate the recording scenario, e.g., room dimensions, walls absorption coefficient, and number of microphones [56]. Nevertheless, we summarize in the following only the core of the angular-spectra based method. For detailed information about its functionalities and implementation, one should refer to the user guide provided with the software.

We describe the use of the MBSS algorithm in three main steps. The first step is to define the possible angles of azimuth and zenith and to assemble the search grid. The program uses elevation instead of zenith, but it can be easily converted: zenith = $\frac{\pi }{2}$− elevation. Based on the grid, the set of possible delays for each pair of microphones is computed, and then, it is resampled to limit the quantity to points in which angular spectrum is calculated. The software offers some options to compute angular spectra. For this work, we used the GCC-PHAT local angular spectra defined as follows [47]:

(15) $$\gamma_{i,j}^{\mathrm{PHAT}}\left(l,f,t\right)=R\left(\frac{{\hat{R}}_{x_{i}x_{j}}\left(l,f\right)}{|{\hat{R}}_{x_{i}x_{j}}\left(l,f\right)|}e^{-2\pi ft}\right)$$

where R is the real operator, *l* is the index of the time frame, *f* is the center frequency of the FFT bin, and *t* is the delay in seconds.

In the second step, the contents of all selected frequency bins are summed up. A linear interpolation is used in $\gamma_{i,j}^{\mathrm{PHAT}}\left(t,l\right)$ to the compute angular spectrum approximation in all possible *t* in each pair of microphones. This value is used to calculate the angular spectrum directly, depending on the direction of arrival, $\gamma_{i,j}\left(l,\phi ,\theta \right)$. Then, angular spectrum of all pairs are summed, generating γ(l,ϕ,θ). For multiple time frames, there are two strategies: sum all time frames or use the maximum overall time frames. The last one is recommended when the signal of interest is only active in a few frames [47]. As the gunshot signals are impulsive, the maximum approach was used and the angular spectrum is then given by the following:

(16) $$\gamma^{\max}\left(\phi ,\theta \right)=\max_{l}\gamma^{\mathrm{PHAT}}\left(l,\phi ,\theta \right).$$

The last step of the MBSS algorithm is a grid search to find the global maximum in the case of only one source or the local maxima when there are multiple sources. If there is only one single source, the DoA angles are obtained from the following:

(17) $$\hat{\phi },\hat{\theta }=\underset{\phi ,\theta }{arg max}\gamma \left(\phi ,\theta \right).$$

### 2.4. Position Estimation

There are a number of ways to estimate the shooter localization. A simple approach uses DoA estimations of muzzle blast from different arrays according, for instance, to the total least squares (TLS) [61] algorithm. Since this method does not use the shockwave component, it can estimate position even of small caliber weapons of which the projectiles do not reach supersonic speed. Another advantage of this method is that, with a sufficient number of arrays, it could be combined with a data-selective algorithm, such as Exhaustive Search, seen in Section 2.3, to remove outliers expected to happen when some arrays do not have a clean sight to the firearm or are heavily affected by multipath [31,32]. On the other hand, in order to use the TLS approach, the system would be more complex and expensive to be deployed, since it requires more than one drone and they need to communicate with the node responsible for the calculation of the shooter’s position with the information of all platforms.

A second approach is to combine shockwave and muzzle blast DoA estimations to compute the probable shooter location [41,62]. As this method uses shockwave components, it is only applicable in the case of supersonic projectiles and when the array is inside the shockwave field of view. Moreover, this method assumes (at least its simplest version) that the projectile has a constant speed, which tends to generate results that overestimate the distance when the shooter is more than 100 m from the array; adaptations are then required to overcome this limitation as stated in Reference [63].

A third approach presented in Reference [24] combines muzzle blast DoA estimation from an elevated array with a digital map containing topographic information to estimate the shooter position. The main concern of this method is to obtain the appropriate digital model of the terrain. As in the TLS approach, this method can estimate the position of subsonic firearms. This approach would be appropriated for our scenario, but focusing on the DoA estimation, we have not carried out a position estimation evaluation in this work.

## 3. System Setup and Signal Acquisition

In this section, we describe the hardware, the drone, and the microphone array used in field recordings. We also provide some information about the shooting site and the environmental conditions the experiment was performed under. We also provide details regarding the data acquisition process, including audio recording and drone flight log data.

### 3.1. UAV and Avionics

We used a DJI Phantom 4. It weights 1.38 kg (battery and propellers included but without the extra hardware used for recording the audio signals) and has a 35 cm diagonal, also featuring a 4K camera, and support for two satellite positioning systems (GPS nad GLONASS). According to the manufacturer [64], the UAV, without any external hardware, is able to resist to wind gusts up to 36 km/h.

The microphone array was mounted in 41 cm metal rods, aligned with the propeller’s arms. The size of the rods was engineered to keep the microphones away from the propellers to reduce the influence of noise caused by air displacement generated by the rotating blades. The four microphones were placed in the same height in a planar structure to avoid interference with the drone’s maneuverability, especially during take off and landing. The planar coordinates for the microphones are given in Table 1, assuming the origin of the coordinate system in the center of the UAV.

**Figure 5:**
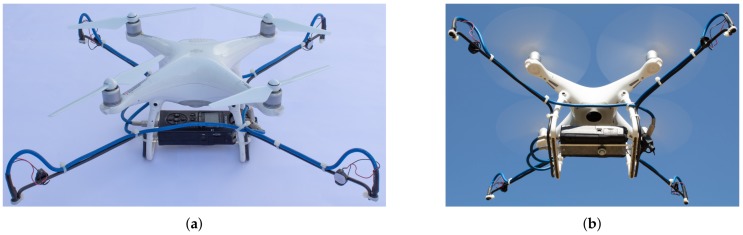
Drone used in the experiments: (**a**) landed; (**b**) during flight.

The gimbal and the camera were removed, allowing the recorder to be placed under the drone (see Figure 5), aligning it with the center of mass of the multirotor, and minimizing the impact on the flight capabilities of the UAV. Care was taken in order not to cover the ultrasonic sensors, located on the underside of the drone’s hull; this would severely affect flight safety and its landing ability.

### 3.2. Environmental Conditions and Shooting Site

**Figure 6:**
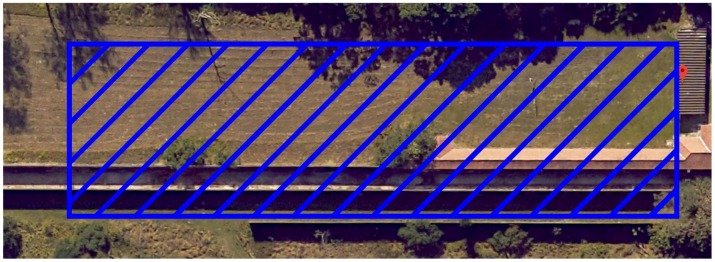
Shooting site: In red (marker) is shooter location, and in blue is the allowed flight zone of the drone. Adapted from Google Maps [65].

The gunshot signals were collected in a shooting site located at the Brazilian Army Evaluation Center (CAEx) on a cloudy day with no strong wind and with a temperature of 24 ${}^{{}^{\circ}}$C. Figure 6 shows a satellite image of the shooting site. The drone’s flight zone was restricted to the blue rectangle of area 30 × 120 square meters to prevent it from flying over sensitive regions and to ensure a clear line of sight to the shooter.

### 3.3. Data Acquisition: Audio and Drone Position and Attitude

The four microphones were connected to a four-channel recorder, TASCAM DR-40 [66], which is convenient given its relatively reduced dimensions and light weight of 0.213 kg without batteries. The TASCAM DR-40 recorder comes with two connectors for external microphones and two built-in microphones, which were rearranged to a single set with four external channels to accommodate four small electret microphones.

**Figure 7:**
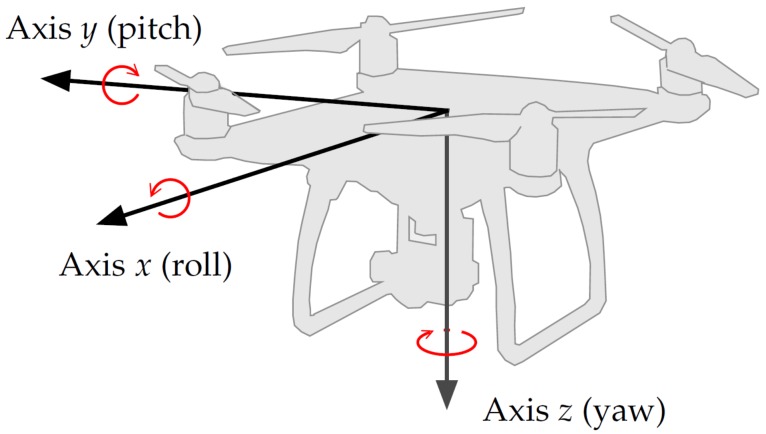
Attitude angles as measured by DJI Phantom 4, adapted from [68].

The recordings were carried out using a sampling frequency of 44.1 kHz and encoded using 24 bits per sample. The drone flight log data was recorded in a file and recovered using *AirData.com* [67]. The log data provides the following information: time (in ms), GPS coordinates (latitude and longitude), altitude, and attitude data (angles yaw, roll, and pitch), as illustrated in Figure 7.

The digital audio recorder and the drone were initialized manually and simultaneously for each flight to synchronize the data about the position and the attitude of the drone with the recorded gunshot signals. As the drone was hovering when the shots were fired, the mismatch due to the manual process is negligible. Furthermore, there was no considerable drift caused by two different clocks, since the battery capacity limits the duration of each flight to a maximum of 18 minutes.

### 3.4. Axis Rotation

The DoA is calculated with respect to the drone’s coordinates of which the axes are not necessarily aligned with the geographic axes. Therefore, after calculating the DoA with respect to the drone’s coordinates, we must rotate the DoA vector in order to match the orientation of the geographic axes. The rotation can be applied by a series of matrix multiplications [69], using the attitude data and the magnetic declination of the location. Considering the axes system shown in Figure 2, the matrix that computes a rotation over axis *z* (yaw-α) is given by the following:

(18) $$R_{z}\left(\alpha \right)=\left[\begin{array}{ccc} \cos(\alpha ) & -\sin(\alpha ) & 0\\ \sin(\alpha ) & \cos(\alpha ) & 0\\ 0 & 0 & 1 \end{array}\right],$$

The rotation matrix over axis *y* (pitch-β) is given by the following:

(19) $$R_{y}\left(\beta \right)=\left[\begin{array}{ccc} \cos(\beta ) & 0 & \sin(\beta )\\ 0 & 1 & 0\\ -\sin(\beta ) & 0 & \cos(\beta ) \end{array}\right],$$

Also, the matrix that performs the rotation over axis *x* (roll-ψ) is given by the following:

(20) $$R_{x}\left(\psi \right)=\left[\begin{array}{ccc} 1 & 0 & 0\\ 0 & \cos(\psi ) & -\sin(\psi )\\ 0 & \sin(\psi ) & \cos(\psi ) \end{array}\right].$$

Therefore, the rotated DoA vector in the geographic coordinate system is expressed as follows:

(21) $$a_{\mathrm{DoA}}^{\mathrm{rotated}}=R_{z}\left(\alpha \right)R_{y}\left(\beta \right)R_{x}\left(\psi \right)a_{\mathrm{DoA}}^{\mathrm{drone}}.$$

Please note that matrix multiplication is not commutative, and therefore, the sequence roll, pitch, and yaw must be respected. Furthermore, the coordinates systems in Figure 2 and Figure 7 are not the same: axes *y* and *z* point in opposite directions; it is necessary to reverse the rotation directions of pitch and yaw angles given by DJI Phantom 4. We must also take into account magnetic declination when rotating over axis *z* Equation (18), or the DoA vector will be aligned with magnetic north instead of geographic north.

## 4. Experimental Results

### 4.1. Simulated Signals

In this work, we used simulated muzzle blast gunshot signals with different noise levels to tune parameters and to evaluate the performance of DoA estimation methods. In order to evaluate the quality of a DoA estimation, we used the angle between the estimated and the actual DoA, herein named angular error and defined as follows:

(22) $$\mathrm{Angular}\;\mathrm{Error}=\cos^{-1}\left(a_{\mathrm{DoA}}^{T}{\hat{a}}_{\mathrm{DoA}}\right),$$

where $a_{\mathrm{DoA}}$ is the correct DoA vector and ${\hat{a}}_{\mathrm{DoA}}$ is the estimated one. Angular error can vary from 0${}^{{}^{\circ}}$, when there is no error in DoA estimation, up to 180${}^{{}^{\circ}}$, when DoA estimation points towards the opposite direction of actual DoA. This metric allows us to compare objectively two different estimations while avoiding distortions in azimuth error when zenith is close to 0${}^{{}^{\circ}}$ or 180${}^{{}^{\circ}}$. We used three performance measures based on angular error to evaluate the DoA estimation methods: mean, standard deviation, and percentage of estimations with angular error less than 3${}^{{}^{\circ}}$. An error of 3${}^{{}^{\circ}}$ is expected to cause an error of approximately 6.28 m at the 120 m range.

The simulation of the muzzle blast signal used 7 real gunshot recordings from a rifle Fz 7.62 M964 (FAL) manufactured by *Indústria de Material Bélico do Brasil* (IMBEL) [70]. Signals were collected with a high-quality microphone in an open and quiet environment, avoiding distortions such as additive noise and multipath propagation effect. These clean gunshot signals were clipped to be 10 ms in length. The selected muzzle blast was considered as the signal of a virtual microphone in the center of the array. Then, we inserted fractional delays to generate each one of the microphone’s target signal, simulating the spatial position of the sound source with respect to the array. Noise was simulated based on eighteen recordings made during flights of the drone with the setup described in Section 3.1. During these recordings, the drone was hovering at different altitude levels, ranging from 10 m to 50 m. At each iteration of the simulation, a random muzzle blast signal and a random noise file were selected. Next, the noise file was clipped at a random point with the size of the desired window.

As the noise may have different magnitudes for each microphone, we define SNR${}_{\mathrm{mean}}$ as the mean SNR across all the sensors:

(23) $${\mathrm{SNR}}_{\mathrm{mean}}=10\log_{10}\left(\frac{1}{M}\left(\frac{\sigma_{s1}^{2}}{\sigma_{n1}^{2}}+\frac{\sigma_{s2}^{2}}{\sigma_{n2}^{2}}+\ldots +\frac{\sigma_{sM}^{2}}{\sigma_{nM}^{2}}\right)\right),$$

where *M* is number of sensors in the array, $\sigma_{ni}^{2}$ is the variance of the noise in the *i*th sensor, and $\sigma_{si}^{2}$ is the variance of the muzzle blast component in the *i*th sensor defined from a 10-ms window.

We divided the results of simulations in two groups: LS method and MBSS, each one having its own parameters to be optimized. In both cases, we studied the effectiveness of preprocessing techniques. In this experiment, we ran 3000 iterations for each SNR value. In each iteration, the DoA was drawn according to a uniform distribution over a semisphere (as already mentioned, we consider that the shooter is in a lower position when compared to the drone).

For the LS method, simulations aimed at the best values of window size (from 20 ms up to 50 ms) and *n* in ES(*n*). As the array is composed of 4 sensors, N=M(M−1)/2=6 pairs of microphones are available, so we tested from n=6 to n=3. Analyzing Table 2, we note that the best estimation was usually obtained using the smallest window. This was expected, since the muzzle blast signal lasts 10 ms and a smaller window would contain less noise without losing information about the muzzle blast signal. As stated in Reference [6], an optimal *n* depends on the SNR: when there is less noise, we should consider more pairs; conversely, when the SNR value gets lower, more pairs have their TDEs compromised and should be discarded. As for the preprocessing techniques, we note that the median filter improves the quality of DoA estimation. However, the Wiener filter implementation used herein did not fit well to the application at hand when combined with the GCC-PHAT. An in-depth analysis using the complete results of the LS simulation in Table A1, would indicate that the median filter has better performance among all estimates with angular errors less than 3${}^{{}^{\circ}}$.

In our simulations, we defined two basic MBSS parameters: grid resolution, which was set to 1${}^{{}^{\circ}}$, and alpha resolution, which was set to 0.5${}^{{}^{\circ}}$. The first one is the minimum increment considered in DoA angles, while the second one is related to the resample of possible delays for each pair of microphones, as mentioned in Section 2.3.2. These parameters do not have a considerable influence on performance with low SNR. Assuming that a muzzle blast would come from below the drone, the search boundaries for azimuth and zenith were set to 0${}^{{}^{\circ}}$ to 359${}^{{}^{\circ}}$ and 90${}^{{}^{\circ}}$ to 180${}^{{}^{\circ}}$, respectively. We explored the most suitable values for window and frame sizes; the former varied from 25 ms up to 50 ms, and the latter varied from 10 ms up to 20 ms.

A summary of the MBSS simulation results containing the best parameters per SNR in relation to the rate of estimations with angular error less than 3${}^{{}^{\circ}}$ are shown in Table 3. We noted that frame-based processing, together with the overall maximum strategy, led to the best performance with a 50-ms window size and a frame size of 12 ms or greater. We also notice that the MBSS method does not work well with the preprocessing techniques previously mentioned. Nevertheless, MBSS proved to be more robust to ego-noise, achieving high hit rates even for SNRs as low as −5 dB. The complete results of MBSS simulation can be seen in Table A2.

Based on the simulation results, we chose 2 schemes to process the real gunshots: MBSS with window size of 50 ms and frame size of 15 ms and LS-method using *n* = 4 and frame size of 20 ms preprocessed with median filtering.

### 4.2. Field Recordings

The recordings were carried out in 5 sets. In the first one, 3 shoots were recorded only to make sure that the system was fully operational and were not used to evaluate its performance. The next four sets contain, respectively, 50, 50, 60, and 87 gunshot recordings. Summing up, we have a total of 250 gunshots, all from a Carbine IMBEL 7.62 IA2 [70]. In each series, the drone’s flight height varied from 8.8 m up to 60.5 m. The upper limit of flight height was set to ensure safety since the additional payload in the drone compromises its ability to withstand wind gusts. In some recordings, both muzzle blast and shockwave components are present, while in cases where the drone was not positioned in the propagation path of the shockwave, only the muzzle blast is present. In this work, we address DoA estimation of the muzzle blast only.

In order to avoid issues related to automatic detection, the system recorded continuously for the duration of the flight, and signals were clipped, manually preserving the muzzle blast only. These two acoustic signatures overlapped in a few recordings. When analysing the results, we found out that azimuth estimations were biased. The bias was similar in the third, fourth, and fifth sets but clearly different for the second run. This, combined with the fact that the UAV required a calibration of its magnetic sensor between the second and the third runs, indicates that this bias can be credited to electromagnetic interference in electronic compassed caused by other circuits aboard. As this bias was spotted only when processing the signals in the laboratory, the value of the compensation had to be estimated directly from the gunshots. To estimate the bias, we computed the mean azimuth error, but in order to mitigate the deleterious effects of possible outliers, we used only DoA estimations with zenith errors less than 3${}^{{}^{\circ}}$. Finally, we obtained bias correction values of −8.6743${}^{{}^{\circ}}$ for the second set and −16.7746${}^{{}^{\circ}}$ for the third, fourth, and fifth sets.

The experiments were designed to evaluate the performed of the algorithms under different controlled values of SNR for the gunshot signals. However, other important measurements from GPS and attitude sensors are assumed to be inherently noisy. Table 4 presents the results obtained for the 247 muzzle blast gunshot signals under test. Although for the simulated signals we used as the hit rate the percentage of shots with angular errors lower than 3${}^{{}^{\circ}}$, for the real gunshot signals, we increased this threshold to 10${}^{{}^{\circ}}$. The results in Table 4 represent an average of the different recording conditions, depending on the position of the drone as shall be seen in the following.

**Figure 8:**
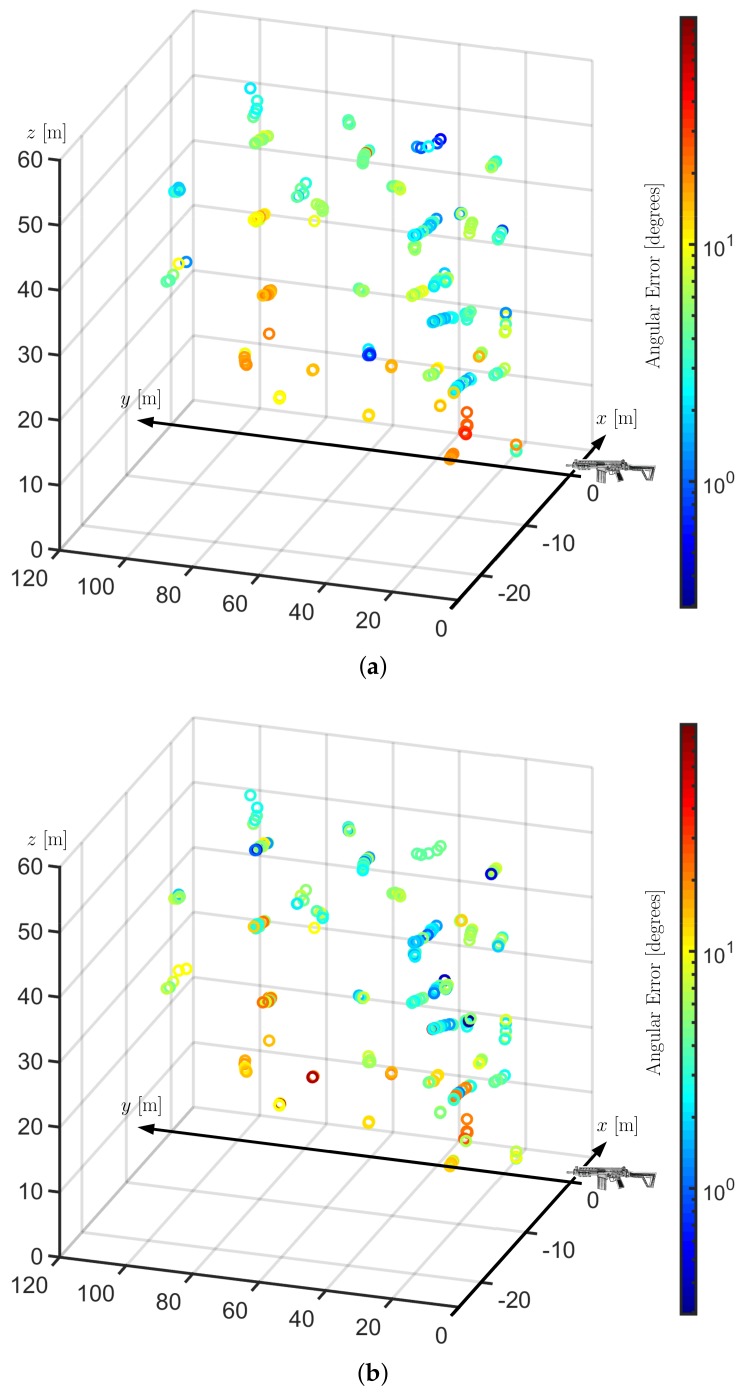
Positions of the drone and respective DoA angular errors: Note that the greater errors (warmer colors) correspond to a region in front of the muzzle. (**a**) Results of the Least Squares method with median filtering. (**b**) Results of the MBSS method without preprocessing.

Figure 8 illustrates the relationship between the position of the drone and the DoA estimation error. Notice that, as the distances between drone and shooter are not substantially large, 120 m at most, the error observed in this experiment is not strongly related to the distance but is rather correlated to relative position: when the drone is within a cone in front of the weapon, the results are poorer. We analyzed the recordings from positions within this cone and observed distorted signals in most of them. These positions are in the field of view of the shockwave but also in the direction of and in a small distance from the gun barrel. This suggests that the causes of the distortions are twofold: overlap of shockwave and muzzle blast components and a great signal intensity saturating the sensor. In an attempt to measure the system performance in better positioning, we took all gunshots recorded in a region outside a 35${}^{{}^{\circ}}$ cone open from the weapon muzzle and the error dropped considerably. The hit rate increased to 92.86% for the MBSS technique and to 95.54% for the LS + MF method instead of the former 72.87% and 70.45%, respectively.

## 5. Discussion and Conclusions

In this work, we analyze the problem of determining the position of a shooter based on gunshot signals acquired using a microphone array mounted on a multirotor UAV. We have conducted a comprehensive literature review on essential topics characterizing the state-of-the-art for this kind of application. We narrow down the focus on the main task, which is to determine the direction of arrival for the muzzle blast and to evaluate the performance of two well-established DoA estimation techniques as well as two important preprocessing methods.

We carry out extensive simulations to evaluate the performance of DoA algorithms and to tune their parameters before finally testing the methods with actual gunshot dates recorded in a firing range. Based on our experimental results, we claim that an aerial microphone array mounted on a drone can be used to obtain good estimates of gunshot direction of arrival using different techniques. The experiments also highlight the fact that the accuracy of the estimates are sensitive to the drone position relative to the shooter and emphasize that better results can be achieved with a system that can fly at higher altitudes, in which case it would be possible to estimate the position of the shooter as well.

Nevertheless, issues like detection, classification, and noise cancellation algorithms require further investigation, testing, and validation to achieve a fully functional, reliable, and autonomous system.

## Figures and Tables

**Table 1:** Planar array coordinates.

Microphone	x (cm)	y (cm)
1	26.5	−25.5
2	26.5	27.0
3	−25.0	26.0
4	−25.0	−25.5

**Table 2:** Least squares (LS) method simulation: The best parameters per signal-to-noise ratio (SNR).

	Without Preprocessing		Median Filter		Wiener Filter	
SNR${}_{\mathrm{dB}}$	*n*/Window Size (ms)	Error < 3${}^{{}^{\circ}}$ (%)	*n*/Window Size (ms)	Error < 3${}^{{}^{\circ}}$ (%)	*n*/Window Size (ms)	Error < 3${}^{{}^{\circ}}$ (%)
10	4/35	99.6000	6/50	99.8000	4/50	88.4333
5	4/20	85.9333	4/20	98.9000	4/50	39.6333
2	4/20	56.9667	4/20	96.7667	4/50	13.7667
0	4/35	35.3667	3/20	89.8000	4/50	6.8000
−2	3/20	19.2000	3/20	74.0333	4/50	2.4667
−3	3/35	13.0667	3/20	59.5333	3/50	1.4333

**Table 3:** Multi-channel Blind Source Separation (MBSS) Simulation: The best parameters per SNR.

	Without Preprocessing		Median Filter		Wiener Filter	
SNR${}_{\mathrm{dB}}$	Window Size/ Frame Size (ms)	Error < 3${}^{{}^{\circ}}$ [%]	Window Size/ Frame Size (ms)	Error < 3${}^{{}^{\circ}}$ [%]	Window Size/ Frame Size (ms)	Error < 3${}^{{}^{\circ}}$ [%]
10	50/12 50/15 50/20	99.4000	50/12 50/15 50/20	98.7333	35/12	92.9667
5	50/12 50/15 50/20	99.1667	50/12 50/15 50/20	98.6667	35/12	63.3667
2	50/12 50/15 50/20	98.7000	50/12 50/15 50/20	98.3000	35/12	29.8000
0	50/12 50/15 50/20	97.6667	50/12 50/15 50/20	96.5667	35/12	15.4000
−2	50/12 50/15 50/20	96.7333	50/12 50/15 50/20	94.6667	35/12	7.5333
−3	50/12 50/15 50/20	95.9333	50/12 50/15 50/20	92.3667	35/12	5.8333
−5	50/12 50/15 50/20	90.7333	50/12 50/15 50/20	78.9333	35/12	3.2667
−8	50/12 50/15 50/20	61.9000	50/12 50/15 50/20	40.2000	35/12	2.1667

**Table 4:** Experimental data of muzzle blast DoA estimation.

	Mean Error (${}^{{}^{\circ}}$)	Standard Deviaton (${}^{{}^{\circ}}$)	Error < 10${}^{{}^{\circ}}$ (%)
LS + MF	8.3823	7.2215	70.4453
MBSS	9.6451	12.2113	72.8745

## Appendix A. Complete Simulation Results

**Table A1:** LS method simulation: complete results.

		Without Preprocessing			Median Filter			Wiener Filter		
SNR${}_{\mathrm{dB}}$	*n*/Window Size (ms)	Mean Error (${}^{{}^{\circ}}$)	Standard Deviation (${}^{{}^{\circ}}$)	Error < $3^{{}^{\circ}}$ (%)	Mean Error (${}^{{}^{\circ}}$)	Standard Deviation (${}^{{}^{\circ}}$)	Error < $3^{{}^{\circ}}$ (%)	Mean Error (${}^{{}^{\circ}}$)	Standard Deviation (${}^{{}^{\circ}}$)	Error < $3^{{}^{\circ}}$ (%)
10	3/20	0.5105	3.9269	99.1000	0.3259	0.5110	99.3000	19.4174	29.2600	63.9667
	3/35	0.4113	2.9569	99.3333	0.3166	0.4897	99.2333	7.5168	19.8348	85.4667
	3/50	0.5498	4.3178	98.8000	0.3137	0.4720	99.3333	6.7859	18.8759	86.3667
	4/20	0.3577	**2.5648**	99.4333	0.2956	0.4566	99.4667	14.2784	25.6210	67.2667
	4/35	**0.3456**	2.5860	**99.6000**	0.2937	0.4598	99.3333	5.7116	17.1664	86.8000
	4/50	0.4492	3.7859	99.5333	0.2983	0.4652	99.4333	**4.9097**	**15.8202**	**88.4333**
	5/20	0.7266	4.2655	97.3333	0.2570	0.4065	99.6000	17.3627	23.8314	49.3333
	5/35	0.6415	4.0465	97.9000	0.2588	0.4150	99.6000	7.2962	17.0812	77.1333
	5/50	0.6343	4.1611	98.0333	0.2599	0.4206	99.6000	6.2528	15.9602	80.3000
	6/20	3.8631	9.2623	79.7000	0.2155	0.4113	99.7667	20.9030	21.0006	31.3000
	6/35	3.6364	8.7809	80.5667	0.2111	0.3641	**99.8000**	11.4076	17.0442	55.8333
	6/50	3.6197	8.7423	80.3000	**0.2081**	**0.3555**	**99.8000**	10.1562	16.0946	59.5667
5	3/20	8.9438	21.7721	83.0333	0.4509	1.8724	98.7667	47.8794	27.9550	13.4000
	3/35	9.0399	21.9193	82.9333	0.3980	0.6057	98.7667	35.8857	31.7066	33.7000
	3/50	9.2772	21.9742	82.3000	0.4750	1.9644	98.1333	33.3909	31.6343	37.4333
	4/20	**6.3045**	18.2118	**85.9333**	0.3558	**0.5525**	**98.9000**	43.6409	28.9848	15.2333
	4/35	6.7532	18.9762	85.2667	0.3634	0.5474	98.8667	32.8974	31.2853	34.8000
	4/50	6.6992	18.5737	84.9667	0.3960	1.0150	98.5000	**30.4495**	31.4588	**39.6333**
	5/20	9.0486	18.0645	68.8333	0.3576	1.0901	98.8867	43.5887	26.2580	7.9333
	5/35	8.9707	18.5345	69.1333	**0.3306**	0.7038	**98.9000**	33.1160	28.5151	23.4333
	5/50	8.7433	17.7275	69.2333	0.3717	1.4043	98.8333	31.2879	28.8127	26.4667
	6/20	16.3355	17.7956	34.0000	0.9664	4.5181	96.1667	42.2534	**22.5981**	3.1000
	6/35	16.1672	17.6804	33.6667	0.6625	2.9858	96.9667	33.7279	23.5199	11.7333
	6/50	15.8325	**17.4014**	34.9000	0.9330	4.5631	96.5667	32.1584	23.7626	13.8333
2	3/20	25.0002	31.2690	54.2333	1.2066	6.5015	95.9667	54.8700	23.4266	2.7333
	3/35	25.1071	31.2757	53.8667	1.2067	6.0516	96.0333	49.7629	27.4016	11.4333
	3/50	25.4807	31.2001	52.8667	1.3870	7.5824	95.3667	48.9076	27.9989	13.2667
	4/20	**20.1555**	28.8475	**56.9667**	**0.8764**	4.7645	**96.7667**	52.9217	23.9341	2.6333
	4/35	20.3873	29.0892	56.7333	0.8803	4.9494	96.7333	48.3238	28.1558	11.7333
	4/50	20.6283	29.0486	56.1000	0.9061	**4.2183**	96.2333	47.3459	28.5305	**13.7667**
	5/20	22.8296	25.8081	35.7667	1.4367	6.0686	93.6667	52.3569	22.8973	0.9667
	5/35	22.7192	26.2225	37.6000	1.4992	6.1723	93.4667	47.8062	26.4787	6.6000
	5/50	22.3695	25.7163	36.5000	1.8749	7.2668	92.2000	46.6417	27.0857	8.6667
	6/20	27.9808	20.9985	12.4000	4.6952	11.2968	80.1000	50.6875	**21.5335**	0.3667
	6/35	27.5228	21.0906	13.3000	4.4991	10.7498	79.8000	46.0894	23.3974	2.3000
	6/50	27.2111	**20.6884**	13.0667	5.6061	12.5749	77.4667	**44.6994**	23.9338	3.7000
0	3/20	36.5372	31.9770	33.4000	3.8697	13.9666	**89.8000**	56.0114	22.1168	0.7667
	3/35	36.5306	31.8651	33.2333	3.7284	13.1700	88.2333	54.1507	24.3032	4.6333
	3/50	36.6136	31.9203	33.6000	5.7540	17.5398	84.9667	52.7311	25.0567	6.1333
	4/20	**31.5958**	31.2282	35.3000	3.6497	13.9061	89.2000	54.8209	22.3306	0.9000
	4/35	32.0653	31.4251	**35.3667**	**3.5862**	**12.6191**	87.9667	53.1904	24.8787	5.0667
	4/50	32.1481	31.5255	35.2333	5.3396	16.2826	83.9333	51.8262	25.7706	**6.8000**
	5/20	32.7753	27.8043	20.3333	5.8269	14.5143	77.2333	54.4921	21.7418	0.1333
	5/35	32.9843	27.8264	18.7000	6.1626	14.3733	75.2000	52.8643	23.9831	2.4000
	5/50	33.8521	28.1587	19.3333	7.4859	16.4049	72.5333	51.4714	24.8103	3.5667
	6/20	34.9195	22.2685	6.1000	11.6739	17.7235	56.8000	53.4600	**21.1648**	0.2333
	6/35	35.2111	**22.2109**	5.6667	12.7202	18.1043	52.5667	50.6964	22.5240	0.7000
	6/50	35.5790	22.4075	5.9000	13.9468	19.1898	50.1667	**49.6944**	22.5882	0.9333
−2	3/20	45.1413	30.2568	**19.2000**	**12.3675**	26.0754	**74.0333**	56.6258	21.7486	0.1333
	3/35	45.9091	29.6356	17.7333	14.7830	28.4758	68.8333	56.1857	22.8085	1.6667
	3/50	45.6604	29.8254	18.3667	17.2721	30.4772	63.3667	55.8175	23.0173	2.2333
	4/20	**42.2719**	30.5784	19.1667	12.4940	25.0592	67.9333	55.8804	21.4336	0.1000
	4/35	42.7540	30.2762	17.7667	14.9671	27.4779	63.3000	55.6017	23.0552	1.9667
	4/50	42.7118	30.1571	17.6000	17.0383	28.8422	58.1333	55.0972	23.3792	**2.4667**
	5/20	42.4860	27.6801	8.8667	15.7599	**23.6227**	51.5667	55.5419	21.4417	0.0667
	5/35	42.6461	27.3037	8.3667	17.8564	25.1710	46.1000	54.9616	22.8000	0.9000
	5/50	43.1371	27.2214	8.2000	19.7907	25.9766	41.5667	54.7407	23.0773	1.0667
	6/20	42.3165	23.0314	2.5333	22.9013	23.6372	30.6000	54.8717	**21.0324**	0.1333
	6/35	42.6043	**23.0215**	2.1333	24.5213	23.9388	26.7333	53.4221	22.0551	0.2333
	6/50	42.6747	23.0524	2.4333	26.4869	24.3458	24.3000	**53.2176**	22.3257	0.3000
−3	3/20	48.9503	28.0092	12.4667	20.0767	32.1463	**59.5333**	56.5733	21.7665	0.1667
	3/35	49.2837	28.3516	**13.0667**	23.4632	34.2915	55.1000	**56.6267**	22.1172	1.0000
	3/50	49.3290	28.3126	12.6000	26.5068	35.6843	49.1000	56.1966	22.5202	**1.4333**
	4/20	47.1451	28.5781	11.9000	**19.7724**	31.0025	53.9333	56.2928	21.4432	0.1333
	4/35	46.7880	28.8198	12.6000	22.9690	32.6417	49.7000	56.4386	22.3254	1.1000
	4/50	47.4005	28.4842	11.5000	26.2416	33.8312	43.3667	55.9051	22.7439	1.3333
	5/20	46.7269	26.2793	5.2000	22.2956	27.3511	38.3000	56.0073	21.2151	0.1333
	5/35	46.3936	26.5745	5.7667	25.1084	29.1954	34.5667	56.1497	22.0678	0.5667
	5/50	46.9904	26.5622	5.7000	28.2202	30.0659	30.0667	55.4949	22.4393	0.6000
	6/20	45.9073	**22.8674**	1.1333	28.5282	24.7190	21.0667	55.4752	**21.0532**	0.1333
	6/35	**45.7383**	22.9903	1.3000	31.0702	25.8381	18.2333	54.8112	22.0272	0.2333
	6/50	46.3164	23.2109	1.1667	32.9346	**25.6870**	15.7667	54.2830	21.9627	0.2667

**Table A2:** MBSS simulation: complete results.

		Without Preprocessing			Median Filter			Wiener Filter		
SNR${}_{\mathrm{dB}}$	Window Size / Frame Size (ms)	Mean Error (${}^{{}^{\circ}}$)	Standard Deviation (${}^{{}^{\circ}}$)	Error < $3^{{}^{\circ}}$ (%)	Mean Error (${}^{{}^{\circ}}$)	Standard Deviation (${}^{{}^{\circ}}$)	Error < $3^{{}^{\circ}}$ (%)	Mean Error (${}^{{}^{\circ}}$)	Standard Deviation (${}^{{}^{\circ}}$)	Error < $3^{{}^{\circ}}$ (%)
10	25/ 10	0.4531	0.4468	99.3000	0.5275	0.6029	98.4333	3.1767	10.7447	90.8000
	35/10	0.4501	0.4328	99.3000	0.5220	0.5896	98.5333	8.9461	20.3771	80.2000
	35/12	0.4473	0.4259	99.3667	0.5094	0.5563	98.6667	**2.2018**	**8.0205**	**92.9667**
	50/10	0.4524	0.4338	99.3333	0.5354	0.6172	98.2333	11.4535	23.4741	77.0000
	50/12	**0.4415**	**0.4084**	**99.4000**	**0.4956**	**0.5301**	**98.7333**	3.3781	11.6631	91.3000
	50/15	**0.4415**	**0.4084**	**99.4000**	**0.4956**	**0.5301**	**98.7333**	3.3781	11.6631	91.3000
	50/20	**0.4415**	**0.4084**	**99.4000**	**0.4956**	**0.5301**	**98.7333**	3.3781	11.6631	91.3000
5	25/ 10	0.5108	0.5392	98.9333	0.6013	0.7111	97.7333	20.9350	28.6107	55.7333
	35/10	0.5069	0.5393	98.8667	0.6015	0.7019	97.8667	31.9753	31.9459	39.9667
	35/12	0.4865	0.4934	99.0667	0.5574	0.6240	98.2333	**16.8103**	**26.6503**	**63.3667**
	50/10	0.4985	0.5100	99.0333	0.5943	0.6907	97.7667	36.2389	31.7327	33.5667
	50/12	**0.4608**	**0.4506**	**99.1667**	**0.5167**	**0.5624**	**98.6667**	23.5154	29.7455	52.9000
	50/15	**0.4608**	**0.4506**	**99.1667**	**0.5167**	**0.5624**	**98.6667**	23.5154	29.7455	52.9000
	50/20	**0.4608**	**0.4506**	**99.1667**	**0.5167**	**0.5624**	**98.6667**	23.5154	29.7455	52.9000
2	25/ 10	0.6587	1.0051	97.1000	0.7438	0.9689	96.1000	40.4421	31.5617	23.8333
	35/10	0.6760	2.3588	97.2667	0.8466	3.2702	96.1333	46.9396	30.9384	16.6333
	35/12	0.5897	0.7246	97.9667	0.6831	0.8328	97.0333	**36.6388**	32.2888	**29.8000**
	50/10	0.6511	1.5885	97.5667	0.8426	3.2499	96.6000	50.0315	**28.4052**	11.7667
	50/12	**0.5097**	**0.5408**	**98.7000**	**0.5769**	**0.6563**	**98.3000**	42.3877	31.8309	22.3000
	50/15	**0.5097**	**0.5408**	**98.7000**	**0.5769**	**0.6563**	**98.3000**	42.3877	31.8309	22.3000
	50/20	**0.5097**	**0.5408**	**98.7000**	**0.5769**	**0.6563**	**98.3000**	42.3877	31.8309	22.3000
0	25/ 10	0.8122	1.9544	95.9000	1.2682	5.4158	93.7333	48.3123	29.8138	12.0667
	35/10	1.1827	5.8337	95.4000	1.6328	8.0185	93.4667	52.3492	27.8695	7.3000
	35/12	0.7021	0.8468	96.5000	0.8722	**1.5264**	95.1667	**45.6052**	30.6252	**15.4000**
	50/10	0.8504	2.5126	95.7667	1.1790	4.3650	93.7667	53.1432	**26.8928**	6.5000
	50/12	**0.5871**	**0.7072**	**97.6667**	**0.8010**	3.4158	**96.5667**	48.8692	30.1058	12.2333
	50/15	**0.5871**	**0.7072**	**97.6667**	**0.8010**	3.4158	**96.5667**	48.8692	30.1058	12.2333
	50/20	**0.5871**	**0.7072**	**97.6667**	**0.8010**	3.4158	**96.5667**	48.8692	30.1058	12.2333
−2	25/ 10	1.9049	9.1781	92.1000	3.6309	14.1082	88.3333	53.3834	27.6259	5.0333
	35/10	2.2230	10.1234	91.9667	4.6370	16.5701	86.6667	55.5020	26.4814	3.5000
	35/12	0.9577	2.8586	94.5333	1.7432	7.3635	91.4000	**51.7095**	29.1208	**7.5333**
	50/10	1.8668	9.2858	93.4000	3.4592	14.0692	89.3000	55.2182	**25.2605**	2.9333
	50/12	**0.6788**	**0.8690**	**96.7333**	**1.2045**	**5.6296**	**94.6667**	53.4769	27.6794	5.8000
	50/15	**0.6788**	**0.8690**	**96.7333**	**1.2045**	**5.6296**	**94.6667**	53.4769	27.6794	5.8000
	50/20	**0.6788**	**0.8690**	**96.7333**	**1.2045**	**5.6296**	**94.6667**	53.4769	27.6794	5.8000
−3	25/ 10	2.7857	12.1656	90.6667	6.7664	20.3681	82.9667	54.2794	27.4513	4.6667
	35/10	3.4863	13.8784	89.6667	8.3755	22.4228	80.2000	56.0700	25.9085	2.4000
	35/12	1.3376	5.6843	92.8667	3.5085	14.0162	87.4667	**52.8001**	28.5094	**5.8333**
	50/10	2.6344	11.6972	91.8000	6.2836	20.0453	84.7333	56.5674	**24.8849**	2.2333
	50/12	**0.9751**	**4.4240**	**95.9333**	**2.0194**	**9.4220**	**92.3667**	54.9000	27.0754	4.2667
	50/15	**0.9751**	**4.4240**	**95.9333**	**2.0194**	**9.4220**	**92.3667**	54.9000	27.0754	4.2667
	50/20	**0.9751**	**4.4240**	**95.9333**	**2.0194**	**9.4220**	**92.3667**	54.9000	27.0754	4.2667
−5	25/ 10	8.9995	23.7791	78.3000	17.8985	32.5969	62.9000	55.5686	26.8271	2.1333
	35/10	12.1111	27.9742	74.6333	22.2779	35.4841	59.0333	57.1703	26.1266	1.1000
	35/12	4.4998	15.6668	84.3000	12.6640	28.4453	69.9000	**54.9973**	27.9755	**3.2667**
	50/10	8.5302	23.3745	80.3000	19.0307	33.8287	64.8667	56.8281	**24.4191**	0.9000
	50/12	**3.0576**	**13.0383**	**90.7333**	**9.1981**	**24.1209**	**78.9333**	56.3419	27.0353	1.8667
	50/15	**3.0576**	**13.0383**	**90.7333**	**9.1981**	**24.1209**	**78.9333**	56.3419	27.0353	1.8667
	50/20	**3.0576**	**13.0383**	**90.7333**	**9.1981**	**24.1209**	**78.9333**	56.3419	27.0353	1.8667
−8	25/ 10	33.5311	39.8177	41.2333	44.3035	40.9917	26.6333	57.2012	26.8990	1.8000
	35/10	36.3345	39.9724	38.0667	46.8466	41.0956	26.0000	57.5182	25.5922	0.8667
	35/12	21.7416	34.7735	53.6667	35.2805	**40.2148**	35.5667	**56.5289**	27.8457	**2.1667**
	50/10	34.0082	39.6064	43.4000	46.5912	40.8337	25.9333	57.7520	**24.7207**	0.6667
	50/12	**18.8424**	**33.5860**	**61.9000**	**34.4365**	40.5661	**40.2000**	57.2580	26.3992	1.7000
	50/15	**18.8424**	**33.5860**	**61.9000**	**34.4365**	40.5661	**40.2000**	57.2580	26.3992	1.7000
	50/20	**18.8424**	**33.5860**	**61.9000**	**34.4365**	40.5661	**40.2000**	57.2580	26.3992	1.7000

## References

[1] Dong J., Wu G., Yang T., Jiang Z. (2019). Battlefield situation awareness and networking based on agent distributed computing. Phys. Commun..

[2] Astapov S., Berdnikova J., Ehala J., Kaugerand J., Preden J.S. (2018). Gunshot acoustic event identification and shooter localization in a WSN of asynchronous multichannel acoustic ground sensors. Multidimens. Syst. Signal Process..

[3] Ramos A.L.L. (2015). Acoustic Sniper Positioning Systems. Ph.D. Thesis.

[4] Kastek M., Dulski R., Trzaskawka P., Piątkowski T., Polakowski H. (2011). Spectral measurements of muzzle flash with multispectral and hyperspectral sensor. International Symposium on Photoelectronic Detection and Imaging 2011: Advances in Infrared Imaging and Applications.

[5] Borzino A.M.C.R., Apolinário J.A., de Campos M.L.R., Biscainho L.W.P. Signal enhancement for gunshot DOA estimation with median filters. Proceedings of the 6th Latin American Symposium on Circuits & Systems (LASCAS).

[6] Borzino A.M.C.R., Apolinário J.A., de Campos M.L.R. (2016). Consistent DOA estimation of heavily noisy gunshot signals using a microphone array. IET Radar Sonar Navig..

[7] Borzino A.M.C.R., Apolinário J.A., de Campos M.L.R. Robust DOA estimation of heavily noisy gunshot signals. Proceedings of the International Conference on Acoustics, Speech and Signal Processing (ICASSP).

[8] Ntalampiras S. (2018). Moving vehicle classification using wireless acoustic sensor networks. IEEE Trans. Emerg. Top. Comput. Intell..

[9] Prince P., Hill A., Piña Covarrubias E., Doncaster P., Snaddon J.L., Rogers A. (2019). Deploying Acoustic Detection Algorithms on Low-Cost, Open-Source Acoustic Sensors for Environmental Monitoring. Sensors.

[10] Prasad R. Alexa Everywhere: AI for Daily Convenience. Proceedings of the Twelfth ACM International Conference on Web Search and Data Mining.

[11] Sibanyoni S.V., Ramotsoela D.T., Silva B.J., Hancke G.P. (2019). A 2-D Acoustic Source Localization System for Drones in Search and Rescue Missions. IEEE Sens. J..

[12] Hoshiba K., Washizaki K., Wakabayashi M., Ishiki T., Kumon M., Bando Y., Gabriel D., Nakadai K., Okuno H. (2017). Design of UAV-embedded microphone array system for sound source localization in outdoor environments. Sensors.

[13] Schmidt R.O. (1986). Multiple emitter location and signal parameter estimation. IEEE Trans. Antennas Propag..

[14] Nakamura K., Nakadai K., Asano F., Hasegawa Y., Tsujino H. Intelligent sound source localization for dynamic environments. Proceedings of the International Conference on Intelligent Robots and Systems (IROS).

[15] Nakamura K., Nakadai K., Asano F., Ince G. Intelligent sound source localization and its application to multimodal human tracking. Proceedings of the International Conference on Intelligent Robots and Systems (IROS).

[16] Okutani K., Yoshida T., Nakamura K., Nakadai K. Outdoor auditory scene analysis using a moving microphone array embedded in a quadrocopter. Proceedings of the International Conference on Intelligent Robots and Systems (IROS).

[17] Furukawa K., Okutani K., Nagira K., Otsuka T., Itoyama K., Nakadai K., Okuno H.G. Noise correlation matrix estimation for improving sound source localization by multirotor UAV. Proceedings of the International Conference on Intelligent Robots and Systems (IROS).

[18] Ohata T., Nakamura K., Mizumoto T., Taiki T., Nakadai K. Improvement in outdoor sound source detection using a quadrotor-embedded microphone array. Proceedings of the International Conference on Intelligent Robots and Systems (IROS).

[19] Wang L., Cavallaro A. Time-frequency processing for sound source localization from a micro aerial vehicle. Proceedings of the International Conference on Acoustics, Speech and Signal Processing (ICASSP).

[20] Wang L., Cavallaro A. (2018). Acoustic sensing from a multi-rotor drone. Sens. J..

[21] Wang L., Cavallaro A. Ear in the sky: Ego-noise reduction for auditory micro aerial vehicles. Proceedings of the 13th International Conference on Advanced Video and Signal Based Surveillance (AVSS).

[22] Basiri M., Schill F., Lima P., Floreano D. (2016). On-board relative bearing estimation for teams of drones using sound. IEEE Robot. Autom. Lett..

[23] Wang L., Sanchez-Matilla R., Cavallaro A. Tracking a moving sound source from a multi-rotor drone. Proceedings of the International Conference on Intelligent Robots and Systems (IROS).

[24] Calderon D.M.P., Apolinário J.A. (2015). Shooter localization based on DoA estimation of gunshot signals and digital map information. Lat. Am. Trans..

[25] Fernandes R.P., Borzino A.M.C.R., Ramos A.L.L., Apolinário J.A. Investigating the potential of UAV for gunshot DoA estimation and shooter localization. Proceedings of the Simpósio Brasileiro de Telecomunicações e Processamento de Sinais.

[26] Fernandes R.P., Ramos A.L.L., Apolinário J.A. Airborne DoA estimation of gunshot acoustic signals using drones with application to sniper localization systems. Proceedings of the SPIE Defense, Security, and Sensing.

[27] Beck S.D., Nakasone H., Marr K.W. An introduction to forensic gunshot acoustics. Proceedings of the 162nd Meeting of the Acoustical Society of America.

[28] Brustad B.M., Freytag J.C. A survey of audio forensic gunshot investigations. Proceedings of the 26th International Conference: Audio Forensics in the Digital Age.

[29] Beck S.D., Nakasone H., Marr K.W. (2011). Variations in recorded acoustic gunshot waveforms generated by small firearms. J. Acoust. Soc. Am..

[30] Routh T.K., Maher R.C. Recording anechoic gunshot waveforms of several firearms at 500 kilohertz sampling rate. Proceedings of the 171st Meeting of the Acoustical Society of America.

[31] Maher R.C. Modeling and Signal Processing of Acoustic Gunshot Recordings. Proceedings of the IEEE 12th Digital Signal Processing Workshop & 4th IEEE Signal Processing Education Workshop.

[32] Maher R.C. Acoustical Characterization of Gunshots. Proceedings of the IEEE Workshop on Signal Processing Applications for Public Security and Forensics.

[33] DuMond J.W., Cohen E.R., Panofsky W., Deeds E. (1946). A determination of the wave forms and laws of propagation and dissipation of ballistic shock waves. J. Acoust. Soc. Am..

[34] George J., Kaplan L.M. (2013). Shooter Localization using a Wireless Sensor Network of Soldier-Worn Gunfire Detection Systems. J. Adv. Inf. Fusion.

[35] Ishiki T., Kumon M. A microphone array configuration for an auditory quadrotor helicopter system. Proceedings of the International Symposium on Safety, Security, and Rescue Robotics.

[36] Strauss M., Mordel P., Miguet V., Deleforge A. DREGON: Dataset and Methods for UAV-Embedded Sound Source Localization. Proceedings of the International Conference on Intelligent Robots and Systems (IROS).

[37] Scalart P., Liu M. Wiener Filter for Noise Reduction and Speech Enhancement. https://www.mathworks.com/matlabcentral/fileexchange/24462-wiener-filter-for-noise-reduction-and-speech-enhancement.

[38] Plapous C., Marro C., Scalart P. (2006). Improved signal-to-noise ratio estimation for speech enhancement. IEEE Trans. Audio Speech Lang. Process..

[39] Ephraim Y., Malah D. (1984). Speech enhancement using a minimum-mean square error short-time spectral amplitude estimator. IEEE Trans. Acoust. Speech Signal Process..

[40] Fitzgerald D. Harmonic/percussive separation using median filtering. Proceedings of the 13th International Conference on Digital Audio Effects (DAFX10).

[41] Mäkinen T., Pertilä P. (2010). Shooter localization and bullet trajectory, caliber, and speed estimation based on detected firing sounds. Appl. Acoust..

[42] Hainsworth S. (2006). Beat tracking and musical metre analysis. Signal Processing Methods for Music Transcription.

[43] Chacon-Rodriguez A., Julian P., Castro L., Alvarado P., Hernández N. (2010). Evaluation of gunshot detection algorithms. IEEE Trans. Circuits Syst. I Regul. Pap..

[44] Freire I.L., Apolinário J.A. Gunshot detection in noisy environment. In Proceeding of the 7th International Telecommunications Symposium.

[45] Ahmed T., Uppal M., Muhammad A. Improving efficiency and reliability of gunshot detection systems. In Proceeding of the 2013 IEEE International Conference on Acoustics, Speech and Signal Processing.

[46] Libal U., Spyra K. (2014). Wavelet based shock wave and muzzle blast classification for different supersonic projectiles. Expert Syst. Appl..

[47] Blandin C., Ozerov A., Vincent E. (2012). Multi-source TDOA estimation in reverberant audio using angular spectra and clustering. Signal Process..

[48] Van Trees H.L. (2004). Optimum Array Processing—Part IV—Detection, Estimation, and Modulation Theory, Optimum Array Processing.

[49] Knapp C.H., Carter G.C. (1976). The generalized correlation method for estimation of time delay. IEEE Trans. Acoust. Speech Signal Process..

[50] Van Den Broeck B., Bertrand A., Karsmakers P., Vanrumste B., Moonen M. Time-domain generalized cross correlation phase transform sound source localization for small microphone arrays. In Proceeding of the 5th European DSP Education and Research Conference (EDERC).

[51] Ribeiro J.G., Serrenho F.G., Apolinário J.A., Ramos A.L.L. (2018). Effective direction of arrival estimation of gunshot signals from an in-flight unmanned aerial vehicle. Autom. Target Recognit. XXVIII.

[52] Qin B., Zhang H., Fu Q., Yan Y. Subsample time delay estimation via improved GCC PHAT algorithm. Proceedings of the 9th International Conference on Signal Processing.

[53] Brandstein M., Silverman H. A robust method for speech signal time-delay estimation in reverberant rooms. Proceedings of the International Conference on Acoustics, Speech, and Signal Processing (ICASSP).

[54] Freire I.L., Apolinário J.A. DoA of gunshot signals in a spatial microphone array: Performance of the interpolated Generalized Cross-Correlation method. Proceedings of the 6th Argentine School of Micro-Nanoelectronics, Technology and Applications (EAMTA).

[55] Ribeiro J.G.C., Serrenho F.G., Apolinário J.A., Ramos A.L.L. Improved DoA estimation with application to bearings-only acoustic source localization. Proceedings of the International Symposium on Signal Processing and Information Technology (ISSPIT).

[56] Lebarbenchon R., Camberlein E. Multi-Channel BSS Locate. http://bass-db.gforge.inria.fr/bss_locate/.

[57] Loesch B., Yang B. (2010). Adaptive segmentation and separation of determined convolutive mixtures under dynamic conditions. International Conference on Latent Variable Analysis and Signal Separation.

[58] Nesta F., Svaizer P., Omologo M. (2009). Cumulative state coherence transform for a robust two-channel multiple source localization. International Conference on Independent Component Analysis and Signal Separation.

[59] Yamaoka K., Ono N., Makino S., Yamada T. Time-frequency-bin-wise Switching of Minimum Variance Distortionless Response Beamformer for Underdetermined Situations. Proceedings of the International Conference on Acoustics, Speech and Signal Processing (ICASSP).

[60] McCowan I., Lincoln M., Himawan I. (2008). Microphone array shape calibration in diffuse noise fields. Trans. Audio Speech Lang. Process..

[61] Doğançay K. (2005). Bearings-only target localization using total least squares. Signal Process..

[62] Freire I.L., Apolinário J.A. (2011). Localização de atirador por arranjo de microfones (in Portuguese). SBAI.

[63] Barger J.E., Milligan S.D., Brinn M.S., Mullen R.J. (2010). Systems and Methods for Determining Shooter Locations with Weak Muzzle Detection. U.S. Patent.

[64] DJI Phantom 4 Specs. https://www.dji.com/phantom-4/info#specs.

[65] Google Google Maps, Map Data: Google. https://www.google.com/maps/.

[66] TASCAM Handheld Recorder DR-40. https://tascam.com/us/product/dr-40/spec.

[67] Airdata UAV. https://airdata.com.

[68] DJI Flight Control. https://developer.dji.com/mobile-sdk/documentation/introduction/flightController_concepts.html.

[69] Rorres C., Anton H. (2010). Elementary Linear Algebra: Applications Version.

[70] Indústria de Material Bélico do Brasil—IMBEL http://www.imbel.gov.br/.

